# Comparison of the NIST and NPL Air Kerma Standards Used for X-Ray Measurements Between 10 kV and 80 kV

**DOI:** 10.6028/jres.105.056

**Published:** 2000-10-01

**Authors:** M. O’Brien, P. Lamperti, T. Williams, T. Sander

**Affiliations:** National Institute of Standards and Technology, Gaithersburg, MD, 20899-8460, USA; National Physical Laboratory, Teddington, Middlesex, UK

**Keywords:** air kerma, free-air ionization chamber, primary standard, reference radiation qualities

## Abstract

A direct comparison was made between the air kerma primary standards used for the measurements of low-energy x rays at the National Institute of Standards and Technology (NIST) and the National Physical Laboratory (NPL). The comparison was conducted at the NPL using NPL reference radiation qualities between 10 kV and 80 kV. The results show the primary air-kerma standards to agree within 0.6 % of their values for beam qualities up to 80 kV.

## 1. Introduction

A direct comparison was made between the air kerma primary standards used for the measurements of low-energy x rays at the National Institute of Standards and Technology (NIST) and the National Physical Laboratory (NPL). The comparison was conducted in June 1998 at the NPL using NPL tungsten reference radiation qualities between 10 kV and 80 kV and the new mammography 28 kV entrance and exit beam offered at NPL. The two NIST primary standards shipped to the NPL for this comparison were the Lamperti (10 kV to 60 kV) and the Ritz (20 kV to100 kV) free-air ionization chambers. Prior to this comparison these primary x-ray standards at these energies have only been compared indirectly through comparisons at the Bureau nternational des Poids et Mesures (BIPM).

## 2. NPL Irradiation Facilities

Two x-ray irradiation laboratories at NPL were used for this comparison. A constant-potential low-ripple generator connected to Machlett^1^ OEG-50A x-ray tubes with either a tungsten or molybdenum anode, each having 1 mm of beryllium inherent filtration, is used to perform calibrations in the NPL low-energy x-ray laboratory. The x-ray tube voltage may be varied from 8 kV to 50 kV in 0.1 kV steps and the tube current is adjustable from 10 μA to 17 mA in 10 mA steps. In the medium-energy x-ray laboratory a constant-potential low-ripple generator connected to either a Philips 160 kV x-ray tube with an inherent filtration of 1 mm beryllium, or a Muller 300 kV tube with an inherent filtration equivalent to 4 mm aluminum, is used to perform calibrations. The voltage may be varied from 50 kV to 300 kV and the current is adjustable from 10 μA to 25 mA. The output of each x-ray system was measured through the use of a transmission monitor chamber. All charge measurements were normalized to the response of the monitor chamber. In the low-energy facility the comparison measurements were made at a distance from the x-ray focal spot of 0.5 m with a field size of 40 mm diameter at the point of measurement. In the medium-energy facility the comparison measurements were made at a distance from the x-ray focal spot of 0.75 m with a field size of 63 mm diameter at the point of measurement. The x-ray beams produced in both NPL calibration facilities are sufficiently uniform to perform primary standard comparisons and calibrations. The NPL reference radiation qualities used for the comparison are listed in Table 1.

## 3. Determination of the Air Kerma Rate

The air kerma rate is determined by the relationship

$$K=\left(\frac{I}{m}\right)\left(\frac{W}{e}\right){\left(1-g\right)}^{-1}\prod k_{i},$$ (1)

where

*I/m* is the mass ionization current as measured by the free-air ionization chamber,
*W/e* is the mean energy per unit charge expended by electrons in dry air with SI unit in joules per coulomb (J/C),
*g* is the fraction of the initial kinetic energy of secondary electrons dissipated in air through radiative processes, but is negligible for x rays with energy less than 300 keV, and
∏*k_i_* is the product of the correction factors to be applied to the free-air ionization chamber.

The physical constants used in the calculation of air kerma follow in Table 2. The calculation of air kerma involves some physical measurements of the primary standards, which are listed in Table 3. The dimensions of the chambers are used to determine the mass of air in which the ionization occurs.

## 4. Characteristics of Air Kerma Standards

### 4.1 Description of Standards

The measurement of the mass ionization current for the determination of air kerma is obtained at both NPL and NIST through the use of primary standard free-air ionization chambers. All four of the free-air ionization chambers used in the comparison are of the conventional parallel plate design. The diameter of the chamber aperture and the length of the collecting region define the mass of air in which the ionization is collected for this type of free-air ionization chamber. The NPL 50 kV chamber is used to measure exposures and air kerma for x rays generated between 8 kV and 50 kV. Reference [1] presents a detailed design description of the NPL chamber and a complete explanation of the correction factors. The NPL 50 kV primary standard has a companion chamber that allows the direct measurement of the air attenuation correction. The air attenuation chamber is of similar construction as the standard, but is fitted with two collector plates separated by the distance equal to the distance between the defining plane of the aperture and the center of the collecting volume of the primary standard. The NPL 300 kV standard is a new chamber which has been designed for use with x rays generated between 40 kV and 300 kV. The design has been described [2], but additional work on the scattered photon correction is continuing and a full report is being prepared. The NIST Lamperti chamber is designed for x-ray exposure and air kerma measurements in the 10 kV to 60 kV region, but is generally used at NIST for the 10 kV and 15 kV qualities. The Lamperti chamber, described in detail in Ref. [3], utilizes a guard-ring system to maintain a uniform electric field. The NIST Ritz chamber, designed for x-ray exposure and air kerma measurements between 20 kV to 100 kV, uses a guard plate and guard strip system to diminish the distortion to the electric fields. The Ritz chamber is described in Ref. [4].

### 4.2 Standard Correction Factors

Although free-air chambers are designed to keep all corrections to the mass ionization current to a minimum, some corrections must be applied. The air attenuation correction, the largest of all the corrections, is the correction for the attenuation of the x rays in the air between the defining plane of the chamber aperture and the center of the collecting volume. The air attenuation correction is expressed by

$$k_{a}=\exp(\mu L)$$ (2)

where *μ* is the air-attenuation coefficient and *L* is the air absorption length, the distance between the defining point of the chamber aperture and the center of the chamber volume.

All of the principal corrections for each chamber are listed in Tables 4 through Table 9. The air attenuation corrections are adjusted to the conditions of 293.15 K and 101.325 kPa. The humidity correction applied to all chambers, as well as its associated uncertainty, was taken from Ref. [5]. No investigation into the polarity effects was conducted for this comparison; previously determined polarity corrections were applied. The NPL 50 kV chamber was designed to achieve no measurable front face penetration. The correction factors for wall transmission and ion loss for the NPL 300 kV chamber are considered to be negligible at the energies used in this comparison. The Lamperti chamber corrections for wall transmission, *k*_p_ and aperture transmission, *k*_1_, are negligible with negligible uncertainties. The Ritz chamber is also considered to have negligible corrections for wall transmission and aperture transmission with relative standard uncertainties of 0.01 % and 0.04 %, respectively. The ion recombination correction for the Ritz chamber was evaluated for an applied potential of 3 kV, less than the routinely used 5 kV.

## 5. Comparison Procedure

The collection of the charge measurements and the positioning of the primary standards was performed by an automated procedure for the majority of the reference radiation qualities used in this comparison. In the NPL low-energy range, the positioning of each standard is accomplished through the use of linear positioning motion controllers and verified manually with micrometers before and after each measurement series. Adjustments in positioning are made to the chamber position since the x-ray tube is held in a fixed position. The typical measurement routine, used in the low-energy NPL calibration facility, involved measuring the air attenuation correction with the NPL air attenuation chamber, measuring the charge with the NPL 50 kV standard, followed by the NIST standard and completed with repeat measurements with the NPL standard and the NPL air attenuation chamber. The complete comparison measurement routine was conducted at least twice for each of the NIST standards.

The positioning routine used for this comparison in the NPL medium-energy facility was not ideal; the process was laborious and imprecise. Due to the size and weight of the NIST standard, the NPL normal calibration positioning procedures were unusable. Since the NIST standard could not be positioned on the same alignment support structure as the NPL chamber, the NIST standard was independently positioned before each measurement. This resulted in a higher positioning uncertainty. The air temperature and pressure for each measurement was monitored using NPL instrumentation in both facilities.

## 6. Measurement Uncertainties

The individual uncertainty components, considered applicable to the comparison, are shown in Table 10 for all of the primary standards. The NIST uncertainties were evaluated according to Ref. [6]. In general, the uncertainties are representative of the uncertainties associated with routine air-kerma measurements at both institutions. The alignment and the charge measurement uncertainties for the NIST chambers result from the NPL alignment and charge collection methods and are different than those for air-kerma measurements conducted at NIST.

## 7. Results and Conclusions

The comparison results are shown in Tables 11 through Table 13 as the ratio of the corrected mass ionization current of the NIST primary standard to that of the NPL primary standard for each reference radiation used in the comparison. The Lamperti chamber response agreed with that of the NPL 50 kV chamber response at the 0.5 % level for the reference radiation qualities produced at 10 kV and at the ±0.1 % level for the reference radiation qualities produced between 11.5 kV and 20 kV. The Lamperti and the NPL chambers have good agreement, considering the uncertainty of this comparison. The Ritz chamber also compared favorably with the NPL 50 kV chamber; agreement was found to be between 0.2 % and 0.6 % for both the tungsten and the molybdenum reference radiation qualities up to 50 kV. The Ritz chamber and the NPL 300 kV chamber compared favorably, 0.1 % and 0.6 %, for 50 kV and 80 kV respectively, despite the alignment difficulties. The Ritz chamber electron loss corrections and the photon scatter corrections are currently being reevaluated for new reference radiation qualities being developed at NIST. Slight changes to the corrections used for 80 kV are expected; however the results for this comparison are based on the correction factors available at the time of the comparison.

## Figures and Tables

**Table 1:** The NPL reference radiation qualities used for the comparison

NPL reference number	Generating potential (kV)	Half-value layer (mm A1)	A1 filtration (mm)	Air kerma rate (mGy s^−1^)

*Tungsten anode*				
2.4.2	10	0.036	0.025	2.1
2.4.3	11.5	0.050	0.050	2.5
2.4.4	14	0.07	0.11	2.2
2.4.5	16	0.10	0.20	2.5
2.4.6	20	0.15	0.30	3.0
2.4.7	24	0.25	0.45	2.2
2.4.8	34	0.35	0.47	3.7
2.4.9	41	0.50	0.56	4.1
2.4.10	44	0.70	0.74	3.7
2.4.11	50	1.00	1.01	3.1
2.2.1	50	1.00	0.75	1.5
RQR6	80	2.9	2.7	1.3

*Molybdenum Anode*				

Entrance	28	0.30	0.03 Mo	0.6
Exit	28	0.62	0.03 Mo	0.02
+ breast phantom				

**Table 2:** Physical constants used in the determination of air kerma

Physical constant	Value	Relative standard uncertainty (%)
*ρ*air^a^	1.293 kg **·** m^−3^	0.01
*W*_air_/*e*	33.97 J C^−1^	0.15
1–*g*_air_	1.0000	0.01

a Density of dry air at *T* = 273.15 K and *p* = 101 325 Pa.

**Table 3:** Dimensions of primary standard chambers used in the comparison

Aperture diameter (mm)	NIST Lamperti	NIST Ritz	NPL 50 kV	NPL 300 kV
Air path length (mm)	39.18	127.39	89.2	493
Aperture diameter (mm)	4.9943	10.0017	8.007	10.014
Applied voltage (V)	1500	3000^a^	1500	3000
Collector length (mm)	10.135	70.03	19.827	100.258
Volume (mm^3^)	198.55	5502.05	998.5	7896
Plate separation (mm)	40	90	62.5	264

a An applied voltage of 5 kV is routinely used at NIST for the Ritz chamber.

**Table 4:** Correction factors used for the Lamperti chamber for the NPL comparison

Correction factor	Generating potential (kV)					Relative standard uncertainty (%)	
	10	11.5	14	16	20	Type A	Type B
Air attenuation *k*_a_^a^	1.0721	1.0520	1.0342	1.0244	1.0169	0.05	0.23
Electron loss *k*_e_	1.000	1.000	1.000	1.000	1.000		0.1
Field distortion *k*_d_	1.000	1.000	1.000	1.000	1.000		0.2
Humidity *k*_h_	0.998	0.998	0.998	0.998	0.998		0.06
Recombination *k*_s_	1.0001	1.0001	1.0001	1.0001	1.0001	0.04	
Photon scatter *k*_sc_	0.9960	0.9962	0.9963	0.9965	0.9966		0.2

a These are nominal values for *T* = 293.13 K and *p* = 101 325 Pa; each measurement is corrected using the air temperature and pressure measured at the collection time.

**Table 5:** Correction factors used for the Ritz chamber for tungsten reference radiation qualities used in the NPL comparison

Correction factor	Generating potential (kV)							Relative standard uncertainty (%)	
	20	24	34	41	44	50	80	Type A	Type B
Air attenuation *k*_a_^a^	1.0557	1.0371	1.0334	1.0253	1.0169	1.0113	1.0054	0.05	0.23
Aperture transmission *k*_l_	1.0000	1.0000	1.0000	1.0000	1.0000	1.0000	1.0000		0.04
Electron loss *k*_e_	1.0000	1.0000	1.0000	1.0000	1.0000	1.0000	1.0012		0.10
Field distortion *k*_d_	1.000	1.000	1.000	1.000	1.000	1.000	1.000		0.20
Humidity *k*_h_	0.998	0.998	0.998	0.998	0.998	0.998	0.998		0.06
Recombinationb *k*_s_	1.0012	1.0010	1.0015	1.0016	1.0015	1.0007 1.0013^c^	1.0007	0.04	
Photon scatter *k*_sc_	0.9939	0.9944	0.9947	0.9950	0.9953	0.9956	0.9965		0.20
Wall transmission *k*_p_	1.0000	1.0000	1.0000	1.0000	1.0000	1.0000	1.0000		0.01

a These are nominal values for *T* = 293.13 K and *p* = 101 325 Pa; each measurement is corrected using the air temperature and pressure measured at the collection time.

b The recombination correction was evaluated for an applied potential of 3 kV.

c Two values are shown for the recombination correction at 50 kV due to the use of different air kerma rates.

**Table 6:** Correction factors used for the Ritz chamber for molybdenum reference radiation qualities used in the NPL comparison

Correction factor	Generating potential (kV)		Relative standard uncertainty (%)	
	28	28	Type A	Type B
	with phantom			
Air attenuation *k*_a_^a^	1.0268	1.0219	0.05	0.23
Electron loss *k*_e_	1.0000	1.0000		0.10
Field distortion *k*_d_	1.000	1.000		0.20
Humidity *k*_h_	0.998	0.998		0.06
Recombinationb *k*_s_	1.0004	1.0002	0.04	
Photon scatter *k*_sc_	0.9945	0.9952		0.20

a These are nominal values for *T* = 293.13 K and *p* = 101 325 Pa; each measurement is corrected using the air temperature and pressure measured at the collection time.

b The recombination correction was evaluated for an applied potential of 3 kV.

**Table 7:** Correction factors for the NPL 50 kV chamber for reference radiation qualities generated between 10 kV and 20 kV

Correction factor	Generating potential (kV)					Relative standard uncertainty (%)	
	10	11.5	14	16	20	Type A	Type B
Air attenuation *k*_a_^a^	1.1749	1.1224	1.0804	1.0570	1.0398	0.05	0.23
Electron loss *k*_e_	1.0000	1.0000	1.0000	1.0000	1.0000		0.006
Field distortion *k*_d_	1.0002	1.0002	1.0002	1.0002	1.0002		0.012
Humidity *k*_h_	0.998	0.998	0.998	0.998	0.998		0.058
Recombination *k*_s_	1.0004	1.0005	1.0005	1.0005	1.0008		0.029
Photon scatter *k*_sc_	0.9949	0.9954	0.9959	0.9962	0.9967		0.115
Polarity effect	1.0004	1.0004	1.0004	1.0004	1.0004		0.023

a These are nominal values for *T* = 293.13 K and *p* = 101 325 Pa; each measurement is corrected using the air temperature and pressure measured at the collection time.

**Table 8:** Correction factors for the NPL 50 kV chamber for reference radiation qualities generated between 24 kV and 50 kV

Correction factor	Generating potential (kV)							Relative standard uncertainty (%)	
	24	34	41	44	50	Mo 28	Mo 28 exit	Type A	Type B
Air attenuation *k*_a_^a^	1.0262	1.0233	1.0169	1.0121	1.0083	1.0231	1.0132	0.05	0.23
Electron loss *k*_e_	1.0000	1.0000	1.0000	1.0000	1.0000	1.0000	1.0000		0.006
Field distortion *k*_d_	1.0002	1.0002	1.0002	1.0002	1.0002	1.0002	1.0002		0.012
Humidity *k*_h_	0.998	0.998	0.998	0.998	0.998	0.998	0.998		0.058
Recombination *k*_s_	1.0007	1.0012	1.0014	1.0013	1.0011	1.0000	1.0000		0.029
Photon scatter *k*_sc_	0.9971	0.9973	0.9975	0.9977	0.9979	0.9972	0.9977		0.115
Polarity effect	1.0004	1.0004	1.0004	1.0004	1.0004	1.0004	1.0004		0.023

a These are nominal values for *T* = 293.13 K and *p* = 101 325 Pa; each measurement is corrected using the air temperature and pressure measured at the collection time.

**Table 9:** Correction factors for the NPL 300 kV chamber for reference radiation qualities generated at 50 kV and 80 kV

Correction factor	Generating potential (kV)		Relative standard uncertainty (%)	
	50	80	Type A	Type B
Air attenuation *k*_a_^a^	1.0401	1.0202	0.20	0.23
Electron loss *k*_e_	1.0000	1.0000		0.006
Field distortion *k*_d_	1.0003	1.0003		0.012
Humidity *k*_h_	0.998	0.998		0.058
Recombination *k*_s_	1.0004	1.0004		0.029
Photon scatter *k*_sc_	0.9908	0.9926		0.115
Polarity effect	1.0004	1.0004		0.023

a These are nominal values for *T* = 293.13 K and *p* = 101 325 Pa; each measurement is corrected using the air temperature and pressure measured at the collection time.

**Table 10:** Applicable relative standard uncertainties for the indicated chamber for the 1998 NPL-NIST comparison, in percent

Source of uncertainty	NIST Lamperti		NIST Ritz		NPL 50 kV		NPL 300 kV	
Combined relative standard	Type A	Type B	Type A	Type B	Type A	Type B	Type A	Type B
Ionization current	0.100	0.017	0.100	0.017	0.100	0.017	0.100	0.017
Volume	0.04	0.01	0.04	0.01		0.155		0.155
Positioning		0.10		0.10		0.10		
Correction factors (excluding *k*_h_)	0.064	0.326	0.064	0.385	0.05	0.267	0.2	0.238
Humidity *k*_h_		0.06		0.06		0.06		0.06
Physical constants		0.15		0.15		0.15		0.15
Quadratic sum	0.13	0.38	0.13	0.43	0.11	0.36	0.22	0.33
Combined relative standard uncertainty for the measurement of uncertainty	0.40		0.45		0.38		0.40	

**Table 11:** Comparison of Lamperti chamber to the 50 kV NPL standard

NPL reference number	Generating potential (kV)	Half-value layer (mm Al)	Ratio of the NIST to the NPL standard chamber response
2.4.2	10	0.036	0.9951
2.4.3	11.5	0.05	1.0006
2.4.4	14	0.07	0.9996
2.4.5	16	0.1	0.9995
2.4.6	20	0.15	0.9992

**Table 12:** Comparison of the Ritz chamber to the NPL 50 kV standard

NPL reference number	Generating potential (kV)	Half-value layer (mm Al)	Ratio of the NIST to the NPL standard chamber response
2.4.6	20	0.15	0.9977
2.4.7	24	0.25	0.9978
2.4.8	34	0.35	0.9989
2.4.9	41	0.5	0.9973
2.4.10	44	0.7	0.9983
2.4.11	50	1.0	0.9983
Mo 28	28	0.30	0.9958
Mo 28 Exit	28	0.62	0.9937

**Table 13:** Comparison of the Ritz chamber to the NPL 50 kV standard

NPL reference number	Generating potential (kV)	Half-value layer (mm Al)	Ratio of the NIST to the NPL standard chamber response
2.2.1	50	1.0	0.9987
RQR6	80	2.9	0.9941

## Footnotes

1 Certain commercial equipment, instruments, or materials are identified in this paper to foster understanding. Such identification does not imply recommendation or endorsement by the National Institute of Standards and Technology, nor does it imply that the materials or equipment identified are necessarily the best available for the purpose.
