# Erratum: Comparison of the NIST and NPL Air Kerma Standards Used for X-Ray Measurements Between 10 kV and 80 kV

**DOI:** 10.6028/jres.105.070

**Published:** 2000-12-01

**Authors:** M. O’Brien, P. Lamperti, T. Williams, T. Sander

**Affiliations:** National Institute of Standards and Technology, Gaithersburg, MD 20899-8460, USA; National Physical Laboratory, Teddington, Middlesex, UK

Correct versions of Tables 11, 12, and 13 are printed in this Errata.

[J. Res. Natl. Inst. Stand. Technol. Volume 105, Number 5, September–October 2000, p. 701]

## Figures and Tables

**Table 11:** Comparison of Lamperti chamber to the 50 kV NPL standard

NPL reference number	Generating potential (kV)	Half-value layer (mm Al)	Ratio of the NIST to the NPL standard chamber response
2.4.2	10	0.036	0.9951
2.4.3	11.5	0.05	1.0006
2.4.4	14	0.07	0.9996
2.4.5	16	0.1	0.9995
2.4.6	20	0.15	0.9992

**Table 12:** Comparison of the Ritz chamber to the NPL 50 kV standard

NPL reference number	Generating potential (kV)	Half-value layer (mm Al)	Ratio of the NIST to the NPL standard chamber response
2.4.6	20	0.15	0.9977
2.4.7	24	0.25	0.9978
2.4.8	34	0.35	0.9989
2.4.9	41	0.5	0.9973
2.4.10	44	0.7	0.9983
2.4.11	50	1.0	0.9983
Mo 28	28	0.30	0.9958
Mo 28 Exit	28	0.62	0.9937

**Table 13:** Comparison of the Ritz chamber to the NPL 50 kV standard

NPL reference number	Generating potential (kV)	Half-value layer (mm Al)	Ratio of the NIST to the NPL standard chamber response
2.2.1	50	1.0	0.9987
RQR6	80	2.9	0.9941

